# 2779. Early Administration of Ceftazidime-Avibactam for Carbapenem-Resistant Enterobacterales Is Associated with Improved Clinical Success

**DOI:** 10.1093/ofid/ofad500.2390

**Published:** 2023-11-27

**Authors:** Ashlan J Kunz Coyne, Kristen Lucas, Elizabeth Shald, M Gabriela Cabanilla, Christine Yost, Sydney VanDorf, Cara Slaton, Wesley D Kufel, James Truong, Justin A Andrade, John Cerenzio, Mark Biagi, Michaela Todd, Bryan White, Joseph Sassine, Jillian E Hayes, Paige Witucki, Carolina Orzol, Mirna Eshaya, Callan Bleick, Kaylee E Caniff, Michael P Veve, Michael J Rybak

**Affiliations:** University of Kentucky, Detroit, Michigan; Wayne State University, Detroit, Michigan; University of New Mexico Hospital, Albuquerque, NewMexico; University of New Mexico Hospitals, Albuquerque, NewMexico; Corewell Health, Royal Oak, Michigan; Corewell Health, Royal Oak, Michigan; Orlando Health, Orlando, Florida; Binghamton University School of Pharmacy and Pharmaceutical Sciences, Binghamton, New York; Brooklyn Hospital, Brooklyn, New York; The Brooklyn Hospital Center, Brooklyn, New York; Brooklyn Hospital, Brooklyn, New York; Swedish American Hospital, Rockford, Illinois; UW Health SwedishAmerican Hospital, University of Illinois at Chicago, Rockford, Illinois; University of Oklahoma Medical Center, Oklahoma City, Oklahoma; University of Oklahoma Health Sciences Center, Oklahoma City, Oklahoma; Duke University Hospital, Durham, North Carolina; Wayne State University, Detroit, Michigan; Anti-Infective Research Laboratory, College of Pharmacy and Health Sciences, Wayne State University, Detroit, Michigan; Anti-Infective Research Laboratory, College of Pharmacy and Health Sciences, Wayne State University, Detroit, Michigan; Anti-Infective Research Laboratory, College of Pharmacy and Health Sciences, Wayne State University, Detroit, MI, Wayne State University School of Medicine, Department of Microbiology and Immunology, Detroit, MI, Detroit, Michigan; Anti-Infective Research Lab, Eugene Applebaum College of Pharmacy and Health Sciences, Wayne State University, Royal Oak, MI; Eugene Applebaum College of Pharmacy and Health Sciences, Detroit, Michigan; Eugene Applebaum College of Pharmacy and Health Sciences, Detroit, Michigan

## Abstract

**Background:**

Carbapenem-resistant Enterobacterales (CRE) are a significant public health threat. Ceftazidime-avibactam (CZA) retains activity against most carbapenemase-producing CRE and demonstrates favorable health outcomes compared to historically best available therapy. While early initiation of effective therapy is critical in serious infections, the impact of time to CZA initiation on CRE infection-related outcomes has yet to be assessed. Aim: to evaluate clinical outcomes of patients with CRE infections receiving early vs. late CZA as the first active β-lactam.

**Methods:**

Multicenter, retrospective cohort of hospitalized adult patients from 2019-2022 with culture-positive CRE and infectious symptoms receiving early vs. late CZA as the first active β-lactam. Early and late CZA were defined as CZA administration within or after 48 hours of index culture collection, respectively. Primary outcome was a composite of clinical success defined as i) the absence of all-cause mortality or microbiological recurrence requiring therapy within 30 days from the end of CZA therapy and ii) continued infectious symptoms during CZA therapy.

**Results:**

In total, 174 patients were included (≤48 hours n=52, >48 hours n=122). Median (IQR) age was 61 (53, 73) years and 56.3% were male. ICU admission rates were similar between early vs. late CZA (73.1% vs. 72.1%; *P*=0.921). Most common infection sources were respiratory (59.8%) and skin and skin structure (9.2%). *Klebsiella pneumoniae* was the most common CRE isolated (66%). The overall median (IQR) of CZA MIC was 2 (1,4). and there was no difference in the receipt of package insert CZA doses in the first 48 hours of CZA therapy between the early vs. late groups, respectively (1.9% vs. 5.2%; *P*=0.311). In total, 40/52 (76.9%) early and 68/122 (55.7%) late CZA patients met the clinical endpoint of clinical success (*P*< 0.001). The early and late CZA groups demonstrated similar ICU length of stay (LOS) (26 [9, 46] vs. 22 [12, 40]; *P*=0.431, respectively).

Table 1. Baseline and Clinical Characteristics

**Figure:**
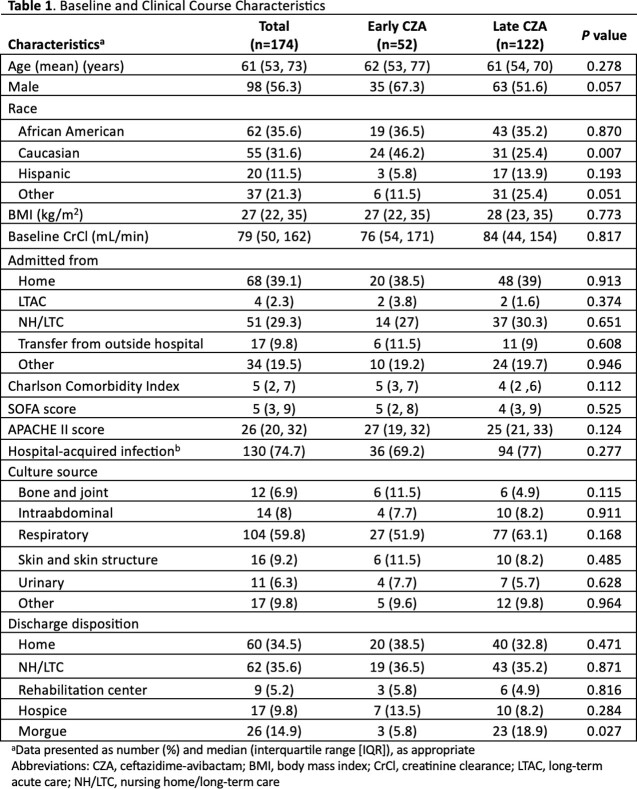

**Conclusion:**

In hospitalized adult patients with CRE infections, CZA administration within 48 hours of culture collection was associated with a higher rate of clinical success compared to CZA administered after 48 hours. Further studies in a larger patient cohort are warranted to verify these results.

**Disclosures:**

**Wesley D. Kufel, Pharm.D., BCPS, BCIDP, AAHIVP**, Merck: Grant/Research Support **Bryan White, PharmD, BCPS, BCIDP**, Gilead Sciences: Advisor/Consultant **Michael J. Rybak, PharmD, PhD, MPH**, Abbvie, Merck, Paratek, Shionogi, Entasis, La Jolla, T2 Biosystems: Advisor/Consultant

